# The Influence of Local Food Environments on Adolescents’ Food Purchasing Behaviors

**DOI:** 10.3390/ijerph9041458

**Published:** 2012-04-16

**Authors:** Meizi He, Patricia Tucker, Jason Gilliland, Jennifer D. Irwin, Kristian Larsen, Paul Hess

**Affiliations:** 1 University of Texas at San Antonio, Department of Health and Kinesiology, One UTSA Circle, San Antonio, TX 78249, USA; 2 School of Occupational Therapy, Rm. 2547, Elborn College, University of Western Ontario, London, ON N6G 1H1, Canada; Email: ttucker2@uwo.ca; 3 Department of Geography, Social Sciences Centre Room 1403, University of Western Ontario, London, ON N6A 5C2, Canada; Email: jgillila@uwo.ca; 4 Faculty of Health Sciences, Arthur & Sonia Labatt Health Sciences Building, Room 215, University of Western Ontario, London, ON N6A 5B9, Canada; Email: jenirwin@uwo.ca; 5 Department of Geography and Program in Planning, University of Toronto, 100 St. George Street, Toronto, ON M5S 3G3, Canada; Email: kristian.larsen@utoronto.ca (K.L.); hess@geog.utoronto.ca (P.H.)

**Keywords:** child and adolescent health, environmental health, nutrition and diet

## Abstract

This study examined the relationship between the neighborhood food environment and the food purchasing behaviors among adolescents. Grade 7 and 8 students (n = 810) at 21 elementary schools in London, Ontario, Canada completed a questionnaire assessing their food purchasing behaviors. Parents of participants also completed a brief questionnaire providing residential address and demographic information. A Geographic Information System (GIS) was used to assess students’ home and school neighborhood food environment and land use characteristics. Logistic regression analysis was conducted to assess the influence of the home neighborhood food environment on students’ food purchasing behaviors, while two-level Hierarchical Non-Linear Regression Models were used to examine the effects of school neighborhood food environment factors on students’ food purchasing behaviors. The study showed that approximately 65% of participants reported self-purchasing foods from fast-food outlets or convenience stores. Close proximity (*i.e.*, less than 1 km) to the nearest fast-food outlet or convenience store in the home neighborhood increased the likelihood of food purchasing from these food establishments at least once per week by adolescents (*p* < 0.05). High fast-food outlet density in both home and school neighborhoods was associated with increased fast-food purchasing by adolescents (*i.e.*, at least once per week; *p* < 0.05). In conclusion, macro-level regulations and policies are required to amend the health-detracting neighborhood food environment surrounding children and youth’s home and school.

## 1. Introduction

Childhood obesity is a burgeoning public health concern worldwide. In Canada, nearly one in three children and youth are either overweight or obese [1,2], with an equally problematic occurrence in the United States [3]. High levels of junk- and fast-food consumption, along with the increase in sedentary behaviors of children and adolescents are considered the leading causes of the dramatic rise in prevalence rates of childhood obesity in recent decades [4].

Children and adolescents may be particularly vulnerable to social and environmental influences that increase the risk of becoming obese. Although children and adolescents can be encouraged to increase their self-control when facing temptation, and can be equipped with knowledge and skills to help understand the context of their life choices, the environments in which they dwell, play, and go to school are linked to behaviors that encourage or discourage healthy bodyweights. In particular, research has identified that the physical environment surrounding children’s and adolescents’ home and schools, including the accessibility and availability of fast-food outlets and convenience stores may negatively impact their food choices [5,6,7].

The inconsistent findings on the relationship between the food environment and eating behaviors warrant further research and investigation. Sturm and Datar examined the relationship between food outlet density and change in body mass index (BMI) over four years in a large cohort of elementary school children using data from the U.S. Early Childhood Longitudinal Study. These researchers found that a higher number of fast-food restaurants per capita was associated with faster BMI gain; however, this finding was not statistically significant [8]. Powell and colleagues has found that greater availability of convenience stores was associated with higher BMI and overweight in youth [9]. A Canadian study found that children in neighborhoods with greater perceived access to “shops with modestly priced fresh produce” had healthier diets and were less likely to be overweight or obese [10]. By contrast, Burdette and Whitaker did not detect any association between overweight and the proximity of fast-food restaurants to their residents in a sample of 7020 preschool children in Ohio [11]. An Australian study even found that the availability of fast-food outlets close to home was associated with lower odds of consuming takeaway or fast-foods among adolescents [12]. This same study also found that the presence of fast-food outlets within 2 km of children’s home further decreased the likelihood of the children being overweight [13].

It has been suggested that increasing consumption of fast-food by children as they enter their teenage years may be due to an increased level of personal autonomy at this critical age; compared to children, adolescents have greater access to their own money and experience greater freedom to make choices about what they consume [14]. The current study examined how adolescents’ food purchasing behaviors are influenced by the food environment around their home and schools. This data is part of a larger, comprehensive study investigating the relationship between the built environment and obesity-related behaviors among adolescents [15,16].

## 2. Experimental Section

This was a cross-sectional study conducted between 2006 and 2007 in London, Ontario, a mid-sized Canadian city of approximately 410,000 people [17]. The London population is predominately white (82%), while average age, income and education attainment are similar to those of the average Ontarian’s profile [17]. This study was approved by the Office of Research Ethics at the University of Western Ontario and the research officers at the two participating school boards. Informed written consent was obtained from both parents and adolescents prior to data collection.

### 2.1. Subjects

Study subjects were students in grades 7 and 8 (aged 11–13 years) from a heterogeneous sample of elementary schools varying by income and neighborhood environment throughout the city of London, Ontario. Of the 51 schools invited, 21 (41%) agreed to participate; 11 from the London District Catholic School Board and 10 from the Thames Valley District School Board. A total of 1666 students were invited to participate; 810 students received parental consent and were present on the day of data collection representing a response rate of 49%. The complete details of the participants and methodology has been published elsewhere [15].

### 2.2. Instruments and Administration

The survey completed by students asked how often they purchased foods from fast-food outlets and convenience stores when with a parent/guardian and also when on their own (including with friends). Specifically, this questionnaire sought information pertaining to four purchasing variables: (1) self fast-food purchasing; (2) fast-food purchasing with parents; (3) Self convenience store food purchasing; and (4) convenience store food purchasing with parents. Fast-food outlets were defined as restaurants where ready to eat foods were ordered at a counter and paid in advance with a list of examples e.g., McDonalds, Burger King, Tim Horton’s, Pizza Pizza, Jack Spratt Subs, A & W, Country Style, Little Caesar’s, Arby’s, Wendy’s, *etc*. Convenience stores were classified as small “variety stores” like Mac’s. This tool was designed specifically for this study by two members of the team and evaluated independently by each other team member to assess the tool’s face validity (*i.e.*, that the questions adequately reflected the study purpose). Following minor revisions to the questionnaire it was then pilot tested with a sample of the target audience to ensure question clarity and comprehension. The survey was self-administered in paper format in classrooms with assistance from trained research staff. A short parental questionnaire was sent home to obtain the demographic characteristics of individual households. The parent questionnaire included questions regarding household address (six-digit postal code) household income, and level of educational attainment of parent(s) or guardian(s). Unique IDs were assigned to child-parent pairs prior to the data collection, which allowed for the linkage of data gathered for each child to additional household data gathered through their parent’s survey.

### 2.3. Food Environmental Parameters

A Geographic Information System (GIS) was used to assess the neighborhood food environment and land use characteristics. Seven hundred and eighty-two out of the 810 (96%) survey respondents reported a valid home postal code, which was “geocoded” to the geographic center of the home postal code using ArcGIS 9.2 (ESRI). Postal codes were used instead of exact home addresses to maintain the anonymity of each respondent. There are 10,714 postal codes in London, and on average, there are 10.4 residences per postal code. Previous research in London and other Canadian cities has suggested that postal codes are a suitable proxy for home addresses [18,19].

Individual home neighborhoods were delineated using a 1 km “straight line buffer” (defined rings of selected radius) around the center point of the postal code of each respondent’s home; school neighborhoods were delineated by creating a 1 km straight line buffer centered on the main entrance of the school. A 1 km distance was chosen for the buffer radius as it is commonly-used in accessibility studies to represent a 10–15 minute walk [20]. Data on fast-food outlets and convenience stores were compiled for 2006 using local business directories (Vernon’s City Directory, *City of London *2006, Vernon Directories Ltd: Hamilton, ON, Canada, 2006) and validated by researchers through telephone calls, field surveys, and inspection of aerial photographs. Fast-food outlets were defined as restaurants where customers ordered at a counter and paid in advance for their food. Convenience stores were classified as small food retailers with a floor area of less than 1000 meters (24-h variety stores, gas stations selling junk foods *e.g*., candies, soda, *etc*).

Data on school locations and parcel-level land use were obtained from the City of London Planning Department. These data were used to calculate two types of “junk food” accessibility measures for each respondent using the Network Analysis functions in GIS: (1) “junk food density”, or the number of fast-food outlets and convenience stores within a 1 km buffer of the students’ home and school; and (2) “junk food proximity”, or the shortest distance from the students’ home and school to the nearest fast-food restaurant and convenience store. The shortest distance between the two locations in question was calculated via the shortest possible path along the City of London’s circulation network, which included roads, trails, and pathways.

Land use mix (LUM) is commonly used to estimate proximity to various destinations. While no clear relationship exists as to how mixed neighborhoods may influence behaviors among adolescents, a connection between land use and health-related activity has been observed in studies of adults [21,22,23]. To calculate LUM, every land parcel within the City of London was classified into six broad classes: recreational; agricultural; residential; institutional; industrial; and commercial; and then the total area of each of the six land uses within each buffer was calculated. Following a methodology used in previous studies [24,25], an entropy index was used to determine LUM within home and school neighborhoods:

           [LUM = −∑*_u_* (p*_u_* ln p*_u_*)/ln *_n_*]           (1)

where *u* is the land use classification; *p* is the proportion of land area dedicated to a particular land use; and *n* is the total number of land use classifications (*i.e.*, six). LUM scores range from zero to one. Zero represents a single land use (e.g., all residential), while a score of one represents even distribution of all six land use classifications.

### 2.4. Data Analysis

Data were entered into SPSS 17.0 (SPSS Inc, Chicago, IL, USA) for statistical analysis. Missing values were excluded listwise. The level of significance for all statistical tests was set at 0.05. Food purchasing behaviors were coded into “less than once per week” or “once per week or more” for each of the four variables (*i.e.*, self fast-food purchasing; fast-food purchasing with parents; self convenience store food purchasing; and convenience store food purchasing with parents). “Once per week or more” was chosen as the cut point as it was considered a “routine” behavioral pattern.

Environmental variables were categorized into distance from home or school to the nearest fast-food outlet or convenience store as “1 km or closer” and “further than 1 km”, as 1 km was considered within walking distance for adolescents [26]. LUM was grouped by quartile. For the home neighborhood environment, LUM cut-offs were: 1st quartile <0.425; 2nd quartile 0.425–0.525; 3rd quartile 0.526–0.629; and 4th quartile >0.629. LUM cut-offs of school surroundings were categorized as: 1st quartile <0.68; 2nd quartile 0.68–0.75; 3rd quartile 0.76–0.78, and 4th quartile >0.78. Number of fast-food outlets within a 1 km buffer of a student’s home postal code or school location was used as an index of fast-food outlet density in each adolescent’s home neighborhood and school environment.

Logistic regression analysis was conducted to assess the influence of the home neighborhood food environment on students’ food purchasing behaviors. The environmental variables, including LUM, distance to the nearest fast-food outlet and convenience store, as well as fast-food outlet density were tested for their relationship to adolescents purchasing behaviors. Since some variables are highly correlated, for example, the “distance to the nearest fast-food outlet” and the “number of fast-food outlets within a 1 km buffer” (r = 0.88), these variables were included in the regression model one at a time. Demographic variables included: grade, gender, and father’s level of educational attainment. Family income was not included in the analysis due to a large number of missing values (40%).

As characteristics of the school neighborhood food environment are system level factors where subjects are nested within clusters (*i.e.*, schools), two-level Hierarchical Non-Linear Regression Models were used to examine the effects of these school-level factors on students’ food purchasing behaviors using HLM (Hierarchical Linear and Nonlinear Modeling version 6.06) software [27,28]. Four models were run separately with the four food purchasing behaviors as the main dependent variables in each model. In the 2-level models, “individual factors” including the student’s gender, grade, father’s education and mode of transportation were considered as first level variables, while “school neighborhood food environment characteristics” as second level variables. A null model, (*i.e.*, random-effects model), comprised of individual students (level 1) nested within a school (level 2), was used to estimate the variance of components of students’ food purchasing behavior at the school level before taking into account of potential predictors.

## 3. Results and Discussion

### 3.1. Results

An even distribution of both male and female students participated, and more grade 8 than 7 students took part in the study. Over 65% of subjects’ fathers had a college degree or higher level of education. Table 1 provides full demographic information.

**Table 1:** Demographic characteristics of study subjects (n = 782)*.

Demographic characteristics	n	Percent (%)
**Gender **		
Boy	371	49.0
Girl	386	51.0
**Grade**		
7	330	42.8
8	441	57.2
**Mode of transportation**		
walking to school	405	52.0
walking from school	466	59.7
**Father’s education**		
high school	245	33.7
college/university	411	56.5
graduate school	72	9.9

* Numbers for each item may total less than total n’s because of missing values.

Approximately 65% of participating students reported buying foods from fast-food outlets or convenience stores while on their own or with friends (Table 2). Over half of students had at least one fast-food outlet within 1 km of their home and, in fact, 28% of students had access to three or more fast-food restaurants within 1 km of their home (Table 3). With regard to convenience stores, 60% of participants had a convenience store less than 1 km from their home (Table 3). Approximately 60% of schools had three or more fast food outlets within a 1 km buffer of their surroundings (Table 3). Those adolescents with fast food outlets within walking distance from their homes (*i.e.*, ≤1 km) were 1.5 times more likely to self-purchase fast-food once per week or more (Table 4). Girls and those in grade 7 were more likely to self-purchase fast-food compared to boys and grade 8 students. In addition, having one or more fast-food outlets within a 1 km buffer in the home neighborhood also increased the chance of self fast-food purchasing by 1.6 times. Participants with a convenience store within 1 km m of their home were 2.5 times more likely to purchase food from these venues than those adolescents who do not have a convenience store within walking distance. Students whose homes fell within the 3rd quartile of LUM (meaning a relatively high, but not the highest LUM) were less likely to purchase foods from convenience stores with parents than those in the bottom quartile (Table 4).

**Table 2:** Food purchasing behaviors of study subjects (n = 782)*.

Food purchasing behaviors	n	Percent (%)
**Self fast-food purchasing**		
Never	276	35.4
1–3 times per month	444	56.9
1–3 times per week	47	6.0
>3 times per week	13	1.7
**Fast-food purchasing with parents**		
Never	176	22.5
1–3 times per month	542	69.4
1–3 times per week	52	6.7
>3 times per week	11	1.4
**Self convenience store food purchasing **		
Never	285	37.1
1–3 times per month	368	47.9
1–3 times per week	87	11.3
>3 times per week	28	3.6
**Convenience store food purchasing with parents**		
Never	402	51.5
1–3 times per month	316	40.5
1–3 times per week	57	7.3
>3 times per week	6	0.8

* Numbers for each item may total less than total n’s because of missing values.

**Table 3:** Home neighborhood and school neighborhood food environment characteristics (n = 782).

Home neighborhood food environment		n	Percent
Number of fast-food outlets within 1 km buffer of student’s home			
	None	353	45.1
	1–2	208	26.6
	≥3	221	28.3
Distance to nearest fast-food outlet from student’s home			
	≤1 km	440	56.3
	>1 km	342	43.7
Distance to nearest convenience store from student’s home			
	≤1 km	462	59.1
	>1 km	320	40.9
LUM quartile	4th (>0.63)	194	24.8
	3rd (0.53–0.63)	198	25.3
	2nd (0.43–0.53)	196	25.1
	1st (<0.425)	194	24.8
**School neighborhood food environment **			
Number of fast-food outlets within 1 km buffer of School			
	None	5	24
	1–2	4	19
	≥3	12	59
Distance to the nearest fast-food outlet from school			
	≤1 km	16	76
	>1 km	5	24
Distance to the nearest convenience store from school		17	81
	≤1 km		
	>1 km	4	19
LUM quartile		5	23.8
	4th (>0.78)		
	3rd (0.75–0.78)	6	28.6
	2nd (0.68–0.75)	5	23.8
	1st (<0.68)	5	23.8

**Table 4:** Home neighborhood food environment and students’ food purchasing behaviors (once per week or more).

Dependent variables	Independent variables ^§^	^#^OR	95% CI	*P*
**Self fast-food purchasing-Model 1**	**Gender ^a^**	1.5	1.4–2.0	0.01
	Girl			
	**Grade ^b^**	0.73	0.53–0.99	0.04
	Grade 8			
	**Distance to fast food outlet ^c^**			
	Less than 1 km	1.5	1.1–2.1	0.01
		R^2^ = 0.04		
**Self fast-food purchasing-Model 2**	**Gender ^a^**	1.5	1.1–2.1	0.03
	Girl			
	**^#^ of fast-food outlets within a 1 km buffer^d^**			
	1–2	1.6	1.1–2.3	0.02
	3 or more	1.7	1.1–2.6	0.009
		R^2^ = 0.04		
Self convenience store food purchasing	**Distance to convenience store ^c^**	2.5	1.5+3.6	0.00
	Less than 1 km			
			R^2^ = 0.05	
Fast-food purchasing with parents	**Gender**	1.8	1.1–3.1	0.04
	Girls			
		R^2^ = 0.02		
Convenience store food purchasing with parents	**LUM**			
	4th quartile	0.97	0.49–1.94	0.96
	3rd quartile	0.39	0.16–0.92	0.03
	2nd quartile	0.68	0.33–1.43	0.32
		R^2^ = 0.04		

^§^ Only significant variables are displayed. In each model, independent variables included demographic variables (grade, gender, and father’s level of educational attainment) and environmental variables (LUM quartile, distance to the nearest fast-food outlet and convenience store and fast-food outlet density). Due to the concern of possible co-linearity, “distance to the nearest fast-food outlet” and “number of fast-food outlets within a 1 km buffer” were separately entered into each model; ^#^ Odds ratio; ^a^ referent = girls; ^b^ referent = grade 7; ^c^ referent ≥ 1 km; ^d^ referent = none; ^e^ referent = bottom quartile.

Multilevel analysis showed that the number of fast-food outlets within a 1 km buffer of the school were positively associated with increased likelihood of students purchasing fast-foods alone (Table 5). Neither the proximity of fast-food outlets nor convenience stores was found to be associated with students’ food purchasing behavior when their parents were around.

**Table 5:** School surrounding food environment and students’ self-fast-food purchasing behaviors (once per week or more).

Model	Variance components	SD	*p*
**Null model** (random effect) Level 2	0.115	0.338	<0.05
			
Multilevel Model*	*Odds ratio ^#^*	**95% CI**	*p*
Gender (1 = boy, 2 = girl)	1.5	1.1–2.0	<0.05
Grade (1 = Grade 7, 2 = Grade 8)	0.7	0.5–0.9	<0.01
Number of fast-food outlets within a 1 km buffer (1 = none, 2 = 1–2, 3 = 3 or more)	1.4	1.1–1.7	<0.05

* The model included level 1 factors (student’s gender, grade, and father’s education and mode of transportation to school) and level 2 factors (school neighborhood food environment characteristics); ***^#^*** odds ratio estimates associated with a unit increase in the predictors.

### 3.2. Discussion

The current study contributes to an understanding of the influence of the neighborhood food environment on adolescents’ food purchasing behaviors. Although self purchasing of junk foods were not very prevalent in this age group, our results suggest that the closer adolescents live to fast-food outlets and convenience stores, the more likely they are to purchase food from these outlets when a parent or guardian is not around. In addition, the greater the density of fast-food outlets in the neighborhood surrounding their home or school, the more likely an adolescent is to purchase fast-food, when a parent or guardian is not present. Furthermore, a relatively high land use mix in the home neighborhood was associated with increased purchasing of food from convenience stores by adolescents with their parents.

It has been suggested that the food environment may play a more important role than individual-level factors on the increasing epidemic of obesity [29]. Research has focused on the influence of the home and school food environment on BMI or eating behaviors among children and adolescents [8,10,11,29,30]. Our study documented and reported the relationship of the objectively-measured neighborhood food environment and food purchasing behaviors from fast-food restaurants and convenience stores in adolescents aged 11 to 13. Self food purchasing behaviors appear not to be very common among the adolescents participating in our study, with approximately 8% of students purchasing fast-foods and 15% purchasing convenience store foods more than once per week. One must note that the current paper reported adolescents’ food purchasing behaviors, not food consumption behavior. The relative low rates of food purchasing by adolescents themselves or with their parents may not necessary reflect low intake of fast foods or junk foods, as they may also eat fast foods or junk foods purchased by parents or guardians. A recent study found a positive association between parents’ fast food purchasing with adolescents’ fast food intake level [31]. Nevertheless, a linkage between food environment and food purchasing behaviors was observed. A close proximity to, and high density of, fast-food outlets was positively associated with increased food purchasing. Previous studies have failed to confirm the potential influence that the neighborhood environment may have on the food choices of adolescents [30]. The lack of findings in previous studies may be due to the complexity and low validity of tools used to measure eating behaviors. As shown in the current study, 60% of schools were surrounded by three or more fast-food outlets within a walking distance. It has been well documented in previous academic studies that fast-food outlets are concentrated within a short walking distance from schools and in disadvantaged neighborhoods [32,33,34]. Repeated exposures to fast-food outlets and convenience stores may encourage children and adolescents to develop unhealthy and unwise purchasing habits which may result in the consumption of poor quality foods as they grow into adulthood. A recent intercept survey with a sample of fourth and sixth graders in the US confirmed that children’s frequent purchased items from corner stores before and after school were energy-dense, low-nutritive foods and beverages, e.g., chips, candy, and sugar-sweetened beverages [35]. Although our participating seventh and eighth graders were yet to be frequent junk food buyers themselves, this potential has serious implications for the consumer habits and nutritional health of future generations. Macro-level regulations and policies are required to mend such health-impeding environments surrounding children and adolescents. 

Consistent with some of the literature on adults [32], the current study showed that a close proximity to fast-food outlets was associated with a high likelihood of adolescents’ fast-food purchases (with parents), further affirming the necessity to regulate the distribution and density of fast-food outlets, especially in disadvantaged neighborhoods and areas immediately surrounding schools [32,33,34]. A relatively high land use mix (the 3rd quartile, but not the upper quartile) seems to decrease the likelihood of adolescents purchasing food from convenience stores when they are with their parents. A high land use mix is an established indication of a walkable neighborhood and thus a physical activity-promoting environment [10]. It is encouraging to find that families living in a relatively diverse land use mix appeared to make infrequent trips to purchase less healthful foods from convenience stores or other unhealthy food retailers. However, it is unclear why decreased food purchasing from convenience stores was only observed in families living in the 3rd, but not the upper quartile of land use mix (*i.e.*, the highest land use mix). It is speculated that families living in the upper quartile may be in a lower income basket than those in the 3rd quartile. As a result, adolescents in the upper quartile may not have sufficient pocket money to make frequent food purchasing from the nearby fast food restaurants or convenience stores. Further investigation is needed to confirm the impact of land use mix on people’s food purchasing behaviors.

Demographic factors, such as adolescents’ gender and grade level, appear to play a role in food purchasing behaviors. Girls were more likely to purchase fast-food by themselves or when they were with parents than boys in this study population. In one regression model, seventh graders appeared more likely to buy fast-foods on their own than eighth graders. Our findings in gender and age difference were inconsistent with the literature. For instance, Neumark-Sztaine *et al*. found no gender differences in *a la carte*, fast food, and convenience store lunch purchases or in snack food vending machine purchases in a cohort of high school American students [36]. This same study also reported older students more likely than their younger peers to make food purchases outside the school premises at fast food restaurants and convenience stores [36]. It is unclear why adolescent girls and seven graders made more food purchasing than boys and eight graders in the current study. Further research is needed to confirm such a phenomenon. 

### 3.3. Limitations

There are a number of limitations in this study. First, this was a cross-sectional study; therefore, we are not able to conclude that the association between the neighborhood food environment and adolescents’ food purchasing behaviors is a causal relationship. Second, our sample participants were drawn from a purposeful selection of schools from varying and diverse geographical areas within the city with respect to socioeconomic status, neighborhood land use, and built form. We were also unable to obtain data and compare the demographic profiles between the participants and non-participants. The current sample did not comprise a random sample of grade 7 and 8 students from London, Ontario, Canada. Nonetheless, the 21 sample schools were dispersed in urban and suburban neighbourhoods throughout the city, the built environment characteristics (e.g., residential densities, land-use mix, and number of food retailers) of the home neighborhoods of participants’ is representative of the full diversity of built environments in the entire city. Third, we did not specifically asked if adolescent participants actually resided within the postal codes designated by the parent participant for the majority of days during the week. In light of different family dynamics that are possible in today’s society e.g., teens that live between households, it may result in inaccuracy in home surrounding food environment assessment. Fourth, the current study used self-reported measures of food purchasing behavior which may be subjected to recall bias. Despite these limitations, this study highlights the relationship between access to fast-food outlets and convenience stores and the food purchasing habits of adolescents.

## 4. Conclusions

In conclusion, a close proximity to fast-food outlets and convenience stores, as well as a high density of fast-food outlets in the home neighborhood increases the likelihood of adolescents purchasing food from these shops. Density of fast-food outlets within a 1 km buffer surrounding school neighborhoods also increased the likelihood of adolescences’ fast-food purchasing. 

This study highlights the needs for macro-level regulations and policies, (e.g., regulating the distribution and density of fast-food outlets) to amend the health-impeding neighborhood food environment surrounding adolescents’ schools, where vulnerable children and adolescents are heavily exposed. School districts may work with local government to develop zoning policies that restrict fast-food establishments near school grounds and enable healthy food providers to locate in school surroundings. Local ordinances could also be implemented to restrict convenience stores and mobile vending in selling calorie dense, nutrition-dense foods and beverages near school grounds.
